# Metagenomic Association Uncovers Host Genotype‐Structured Rhizobacterial Networks and Novel Taxa That Enhance Soybean Salt Tolerance

**DOI:** 10.1002/advs.76373

**Published:** 2026-07-13

**Authors:** Yu Luo, Feng‐Li Kang, Qi‐Min Li, Wei‐Cai Yang

**Affiliations:** ^1^ Institute of Genetics and Developmental Biology Chinese Academy of Sciences Beijing China; ^2^ School of Life Sciences Yunnan University Kunming China; ^3^ University of Chinese Academy of Sciences Beijing China

**Keywords:** metagenomic association, plant growth‐promoting rhizobacteria (PGPR), rhizosphere microbiome, salt tolerance, soybean, *Thalassospira xiamenensis*

## Abstract

Salinity is an escalating agricultural challenge, yet plant microbiomes offer a promising avenue for improving salt tolerance. Nevertheless, most naturally occurring microbes remain unevaluated for plant growth–promoting function, and systematic approaches to uncover salt‐tolerance‐enhancing plant growth–promoting rhizobacteria (PGPR) are limited. Here, using soybean as a model, we implement a quantitative framework to characterize rhizosphere microbial networks and nominate novel taxa functionally associated with plant salt tolerance. We introduced a salt tolerance index (STI) to quantify plant salt tolerance and normalize performance across heterogeneous natural soil salinity. Metagenomic sequencing and co‐occurrence analysis revealed distinct rhizosphere microbiota structures between tolerant and susceptible soybeans. In tolerant soybeans, *Pseudomonas* dominated as the hub of a highly interconnected network, whereas susceptible accessions showed a fragmented network dominated by *Acinetobacter*. Correlation analyses identified bacterial taxa positively associated with STI, including documented salt‐tolerant PGPR and novel candidates. Greenhouse experiments showed that one candidate, *Thalassospira xiamenensis*, enhances soybean salt tolerance and reshapes host ion‐transport and oxidative‐stress gene expression under salinity, validating our screening strategy. Our culture‐independent metagenomic association approach reveals host genotype–structured rhizosphere microbial networks underlying salt tolerance and provides an efficient, labor‐saving means for high‐throughput identification of salt‐tolerant PGPR.

## Graphical abstract

An STI‐guided metagenomic association framework links soybean salt‐tolerance phenotypes with rhizosphere microbiome composition and network organization in natural saline soils. The approach identifies candidate beneficial taxa, including *Thalassospira xiamenensis*, and provides an efficient strategy for mining stress‐protective microbes from native rhizosphere communities.

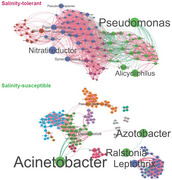

## 1 Introduction

Soil salinization is among the most serious threats to agricultural productivity and food security worldwide [1]. In coastal farmland, salinity is increasingly intensified by sea‐level rise and saltwater intrusion, with NaCl as the dominant soluble salt limiting plant growth [2, 3]. More generally, salinity in agricultural soils can also be intensified by poor‐quality irrigation water, drought, excessive fertilizer input, and inadequate drainage. Over the past decade, the extent of salt‐affected land has increased from 6% to 7% of the Earth's total land area, now approaching 1 billion hectares [3, 4]. Coastal saline soils therefore represent both a major agronomic challenge and a distinctive ecological setting for studying crop adaptation to salinity. However, salinity in coastal soils is inherently heterogeneous because of shallow saline groundwater, evaporation, and related hydrological processes, complicating field‐based phenotypic comparisons in such landscapes [5, 6].

At the physiological level, salt stress is driven mainly by sodium and chloride ions and imposes three major constraints on plants: osmotic stress, ionic stress, and secondary oxidative stress [7, 8]. To cope with these stresses, plants deploy a range of adaptive responses, including stomatal closure, osmoprotectant biosynthesis, Na^+^ exclusion, ion compartmentalization, and detoxification of reactive oxygen species (ROS) [3]. Despite major progress in identifying salt‐responsive genes and pathways, relatively few have translated into practical strategies for improving crop performance in saline soils [4].

Rhizosphere microorganisms are increasingly recognized as important contributors to plant performance under abiotic stress [9]. Plants actively shape rhizosphere microbiota through root exudates, which have been estimated to account for a substantial fraction of photosynthetically fixed carbon [9]. Plant growth‐promoting rhizobacteria (PGPR) can enhance plant salt tolerance by improving ionic and nutritional homeostasis through the production of volatile organic compounds (VOCs), antioxidants, osmolytes, phytohormones, and extracellular polymeric substances (EPS) [10]. These microbial functions can promote ion buffering, root water uptake, and nutrient availability under saline conditions. Natural saline soils are therefore likely to represent an underexplored reservoir of stress‐adapted microbial taxa and consortia with potential to improve crop salt tolerance.

At the same time, rhizosphere assembly is shaped not only by soil conditions but also by host genotype. Root exudate composition is a key part of this effect, as root‐derived metabolites, including flavonoids, strigolactones, organic acids, amino acids, and other specialized compounds, can act as microbial chemoattractants or repellents [11, 12, 13]. Beyond exudate chemistry, host genetic effects also involve nutrient‐sensing, stress‐response, and immune pathways that contribute to rhizosphere community assembly. Notably, these host influences extend beyond species‐level differences, and even single‐gene changes can markedly alter rhizosphere community composition [14]. In rice, NRT1.1B/OsNPF6.5, encoding a nitrate transporter/sensor, affects root microbiota composition and nitrogen use [15]. In Arabidopsis, phosphate‐starvation regulators PHR1/PHL1 and the PHR1–RALF–FERONIA module connect phosphate stress, immunity, and root microbiome assembly [16, 17], while the root‐specific transcription factor MYB72 shapes the microbiome through coumarin exudation [18]. More recently, wild soybean was shown to enrich beneficial root‐associated Pseudomonas under salt stress through purine, especially xanthine, exudation [19]. Together, these findings support the view that the plant and its associated microbiota form a functional holobiont that contributes to plant health and resilience [20]. The concept of hologenome breeding highlights a potential route for crop improvement by targeting plant genetic traits that shape rhizosphere microbiomes and their ecological functions, although microbiome‐mediated traits such as salinity tolerance remain largely underexplored in conventional breeding strategies [21]. Against this background, an important question is whether genotype‐dependent differences in host salt tolerance are associated with shifts in rhizosphere microbiome composition and ecological network architecture under natural saline conditions.

A widely used approach for identifying PGPR involves isolating bacterial strains from the rhizosphere or diverse stress‐associated environments and subsequently evaluating their plant growth‐promoting traits in vitro or *in planta*. The advent of high‐throughput sequencing enabled culture‐independent profiling of root‐associated microbiota, most prominently via 16S rRNA gene amplicon sequencing, which can be integrated with culture‐dependent approaches to improve the identification and characterization of candidate PGPR [22]. Systematic, high‐coverage cultivation strategies for plant‐associated microbiota, coupled with whole‐genome sequencing of isolates, were developed to enhance reconstitution efficiency and facilitate mechanistic investigations [23, 24]. Culture‐based investigations have identified a recurrent set of rhizosphere genera, such as *Bacillus*, *Pseudomonas*, *Enterobacter*, *Streptomyces*, *Klebsiella*, and *Ochromobacter*, as key promoters of crop growth and productivity in saline environments [19, 25]. In addition to salt stress, *Pseudomonas* has been shown to enhance plant resistance to aluminum toxicity, pathogens, and desiccation [14, 26, 27]. However, much of the microbial diversity and community‐level interactions in natural saline soils remain poorly explored, and high‐throughput strategies for systematically identifying salt‐tolerant PGPR are still limited. Integrated approaches linking field‐based phenotyping under heterogeneous saline conditions with rhizosphere microbiome analysis are also lacking. It therefore remains unclear whether genotype‐dependent differences in soybean salt tolerance are associated with distinct rhizosphere microbiome composition and network organization under natural saline conditions, beyond what can be explained by the salinity gradient alone.

Soybean is suitable in this context because it is a globally important oil‐ and protein‐producing legume, relevant to coastal saline agriculture, with additional value for the utilization and improvement of nutrient‐poor marginal soils through symbiotic nitrogen fixation. Meanwhile, shotgun metagenomic sequencing has proven to be a robust quantitative tool for detecting and profiling microorganisms in a variety of biological and ecological settings [28]. This approach minimizes amplification bias and copy‐number distortion, enables simultaneous profiling of organisms across multiple microbial kingdoms, enhances detection of low‐abundance taxa, offers high species‐level resolution, and directly links community composition to functional gene and pathway annotations [29, 30]. Here, we applied a metagenomic association framework to examine genotype‐associated rhizosphere microbial networks in soybean and to identify novel PGPR taxa linked to salt tolerance. As a first step, we leveraged natural salinity heterogeneity in coastal saline soils to develop a salt tolerance index (STI) that standardizes salt tolerance across genetically diverse soybean accessions. By combining STI‐based phenotyping with shotgun metagenomic profiling of rhizosphere communities from representative salt‐tolerant and salt‐susceptible accessions, we found that tolerant genotypes recruited densely connected microbial networks with Pseudomonas as a central hub, whereas susceptible genotypes harbored more fragmented networks enriched in genera such as Acinetobacter. Association analyses between STI and microbial abundance identified a set of bacterial genera and species positively linked to soybean salt tolerance, many of which have not previously been implicated in this trait. Among these, Thalassospira xiamenensis was experimentally validated to enhance soybean growth and nitrogen fixation under salt stress and to modulate host ion‐transport and redox‐related gene expression. Our work not only identifies new microbial contributors to soybean salt tolerance and reveals their associated microbial networks but also demonstrates an efficient strategy to mine native rhizosphere communities for microbes with plant stress‐protective traits.

## 2 Results

### 2.1 Soybean Accessions Show Genotype‐Associated Differences in Salinity Tolerance

**FIGURE 1:**
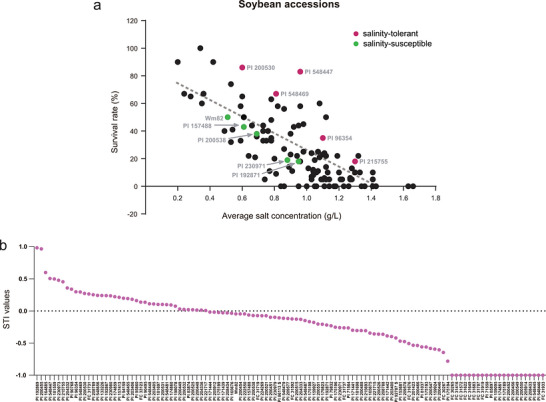
Classification of soybean salt tolerance using the STI. (a) Survival rate (%) of soybean accessions (*n* = 118) exposed to saline conditions in the YRD, plotted against average local soil salinity (g/L). Each point represents one accession (∼40 plants in square‐block plots; salinity measured for each live and dead plant and averaged per accession). Accessions were classified as salinity‐tolerant or salinity‐susceptible by their position relative to the fitted regression (above = tolerant; below = susceptible): Survival rate (%) = 85.69−60.71 × Salt concentration (g/L). Accessions selected for rhizosphere shotgun metagenomics are highlighted (tolerant = red; susceptible = green). These accessions were selected to represent contrasting STI‐defined salt‐tolerance categories while spanning a broad range of local soil salinity conditions, thereby reducing salinity‐range bias in downstream rhizosphere microbiome comparisons. (b) Distribution of STI values for the same accessions. STI normalizes performance to local salinity, enabling between‐genotype comparisons under heterogeneous conditions (STI > 0 indicates better‐than‐expected/tolerant; STI < 0 indicates worse‐than‐expected/susceptible; STI = 0 equals the model prediction; STI = −1 denotes no survival). The assay and STI computation were replicated at the same site in the subsequent year with similar results.

To identify core microbiota and keystone taxa associated with salt‐tolerant soybean accessions and to discover novel salt‐tolerant PGPR, we first assessed the salt tolerance of 118 genetically diverse soybean accessions grown in natural coastal saline soils (Table S1). However, the highly patchy salinity mosaics that characterize natural coastal saline soils hinder straightforward screening for soybean salt tolerance. To correct for this spatial heterogeneity, we plotted local soil salinity against the survival rate of each soybean accession (Figure 1a). Twenty‐two accessions exhibited complete mortality and were excluded from subsequent regression analysis, because zero‐variance responses disproportionately bias the intercept and violate linear‐model assumptions. For the remaining 96 accessions, an ordinary least‐squares (OLS) regression yielded: Survival rate (%) = 85.69−60.71 × Salt concentration (g/L), with an adjusted *R* ^2^ = 0.52 and overall significance *p* < 2.2 × 10^−16^ (Figure 1a and Figure S1a). A non‐parametric LOESS smoother nearly coincided with the OLS line, confirming an approximately linear decline in survival with increasing field salinity (Figure S1a). To ensure that the fitted linear model faithfully represents the observed decline in soybean survival with rising field salinity, we evaluated its assumptions with complementary graphical and formal diagnostics. The Ramsey RESET test (*p* = 0.27) detected no unmodelled non‐linear structure, consistent with the random scatter about zero in the residuals‐vs.‐fitted plot (Figure S1b). The Q–Q plot of model residuals indicated an approximately normal distribution, with points closely following the theoretical line except for a minor deviation at the upper tail (Figure S1c). The approximately flat trend in the scale‐location plot is consistent with the non‐significant Breusch‐Pagan result (*p* = 0.21), supporting homoscedasticity (Figure S1d). Finally, every observation falls below the conservative Cook's‐distance threshold of 0.5 in the residuals‐vs.‐leverage plot, indicating no influential points (Figure S1e). Collectively, these diagnostics confirm that the survival‐salinity relationship is well captured by a simple linear model, suggesting that field salinity is the primary determinant of soybean survival. Accordingly, we operationally defined accessions that fall above the fitted regression line as salt‐tolerant and those below it as salt‐susceptible. Because soil salinity varies across the field, direct comparisons of salt tolerance across accessions are inappropriate. We therefore normalized salt‐tolerance performance by transforming each accession's observed survival (*Y_i_*) and its model‐predicted survival (*Ŷ_i_* = 85.69−60.71∙*X_i_*) into a symmetric, bounded (‐1 to 1) STI value (*STI_i_* = (*Y_i_* − *Ŷ_i_*) / (*Y_i_* + *Ŷ_i_*)). Accessions with positive STI values, indicating better‐than‐predicted survival, were classified as salt‐tolerant, whereas those with negative values were deemed salt‐susceptible. Greater STI values denote higher tolerance, and an STI of ‐1 corresponds to accessions with no plants survived (Figure 1b and Table S1). Our analyses demonstrate that field salinity is the dominant ecological determinant of survivorship across soybean accessions in the heterogeneous coastal landscape and generates STI values that standardize salt‐tolerance measurement for each accession.

### 2.2 Genotype–Microbiota Interactions Restructure Rhizosphere Communities and Enrich Energy and Detoxification Functions in Salt‐Tolerant Soybean

**FIGURE 2:**
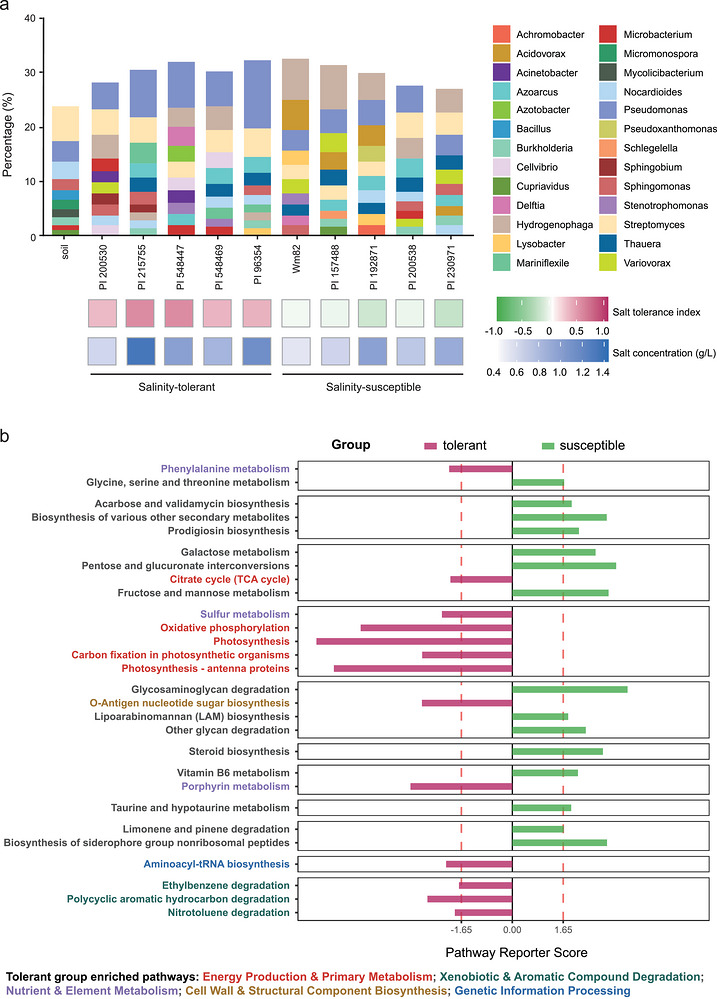
Dominant genera and functional shifts in tolerant vs. susceptible rhizospheres. (a) Top 10 microbial genera (by relative abundance) in bulk soil and in ten soybean accessions (5 salt‐tolerant, 5 salt‐susceptible) profiled by shotgun metagenomics; heatmaps beneath each accession show local soil salinity and STI. For each soybean accession, three biological replicate rhizosphere samples were collected and processed for DNA extraction. (b) KEGG pathway enrichment for salt‐tolerant vs. salt‐susceptible groups, shown as reporter scores (pathway‐level Z‐scores aggregating KEGG Orthology‐wise differential signals; positive = enriched in tolerant, negative = enriched in susceptible). Dashed lines mark |reporter score| = 1.65, the significance cutoff (∼5% one‐sided under a normal approximation).

Salt tolerance variation among soybean accessions can reflect both plant genotype and the root microbiota, which is itself shaped by plant genotype and environmental conditions. To examine genotype–microbiota interactions while reducing confounding by salinity‐driven microbial effects, we selected five tolerant and five susceptible accessions grown across a broad salinity range (Figure 1a and Table S1). Using multiple tolerant and susceptible accessions increased host‐associated variation in the microbiome comparisons, reduced the influence of any single accession‐specific effect, and improved our ability to identify candidate PGPR with broader compatibility across soybean backgrounds. To characterize the rhizosphere microbiomes of these soybean accessions, we sampled phenotypically uniform plants for each accession, isolated their rhizosphere microbiota, and subjected the samples to shotgun metagenomic sequencing. Across bulk soil and rhizosphere samples, the metagenomes contained 8390 species‐level taxa, comprising 90.9% Bacteria, 4.7% Archaea, 2.7% Viruses, and 1.7% Fungi (Figure S2). We then quantified variation in microbial abundance and composition across accessions and bulk soil based on these metagenomes. To reduce the confounding influence of nodulation‐driven taxa, we excluded the principal soybean rhizobial genera (Sinorhizobium, Ensifer, Rhizobium, Bradyrhizobium, and Mesorhizobium), together with Agrobacterium, before downstream analyses. These taxa can show notably uneven distributions across accessions and biological replicates because they more directly reflect localized symbiotic or opportunistic colonization and, as some of the most abundant taxa in the dataset, can substantially bias relative‐abundance profiles and obscure patterns among the remaining taxa. After exclusion, the relative abundances of the remaining taxa were renormalized to 100% for downstream analyses, so downstream analyses reflect the remaining rhizosphere community. Bulk soil was dominated by *Streptomyces*, with *Pseudomonas* and *Nocardioides* ranking second and third (Figure 2a). Salt‐tolerant soybean roots shifted the community toward *Pseudomonas* dominance and uniquely recruited *Cellvibrio* and *Acinetobacter* among the ten most abundant genera. Pseudomonas is well known for mitigating plant salt stress [19], whereas Acinetobacter includes endophytic strains that enhance plant growth [31]. In contrast, four of five susceptible accessions were dominated by *Hydrogenophaga*, and their top‐ten lists uniquely featured *Acidovorax*, which spans plant pathogens and root‐associated commensals [32]. Regardless of host phenotype, *Hydrogenophaga*, *Thauera*, and *Azoarcus* were enriched relative to bulk soil. *Hydrogenophaga* are hydrogen‐oxidizing Betaproteobacteria found in plant‐associated niches, yet in‐planta evidence for salinity‐tolerance promotion is lacking. Members of Azoarcus have been characterized as grass endophytes with nitrogen‐fixing capacity [33]. The dominant rhizobacterial genera correlate closely with soybean salt‐tolerance scores yet show little relation to ambient soil salinity (Figure 2a). Overall, accessions enriched for genera characteristic of salt‐tolerant soybeans performed better under salt stress, supporting host genotype–microbiota interactions as a primary driver of differences in dominant rhizosphere taxa, with ambient salinity contributing comparatively little.

Beyond taxonomic composition, we compared rhizosphere functional gene repertoires in salt‐tolerant and susceptible accessions using Kyoto Encyclopedia of Genes and Genomes (KEGG) annotations. KEGG enrichment analysis revealed distinct functional profiles between tolerant and susceptible groups (Figure 2b). The tolerant rhizosphere microbiomes were enriched for energy‐intensive pathways, sulfur assimilation, porphyrin metabolism and aromatic‐compound degradation, indicating greater respiratory and detoxification capacity, with aminoacyl‐tRNA biosynthesis and O‐antigen nucleotide‐sugar biosynthesis supporting active protein synthesis and cell‐surface remodeling. By contrast, the susceptible rhizosphere microbiomes were enriched for sugar metabolism, glycan degradation and the production of specialized secondary metabolites, suggesting a community with reduced emphasis on core energy metabolism and a shift toward carbohydrate turnover and secondary pathways. Overall, the functional partitioning of rhizosphere microbiomes mirrors host salt tolerance, with tolerant communities configured for energy‐demanding stress mitigation and susceptible communities oriented toward carbon processing and ancillary metabolism.

### 2.3 Co‐Occurrence Network Topology Reveals Pseudomonas‐Centric Cooperative Microbiomes in Salt‐Tolerant Soybeans

**FIGURE 3:**
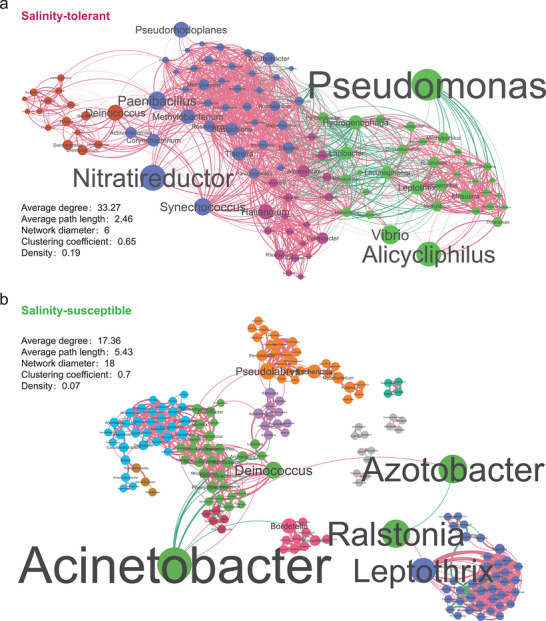
Co‐occurrence networks among genera in salt‐tolerant and salt‐susceptible rhizospheres. Co‐occurrence networks built from genera‐genera associations (Spearman correlation; taxa with cumulative relative abundance ≥ 0.005; |*ρ*| ≥ 0.7; BH‐adjusted *p* < 0.05) are shown for (a) salt‐tolerant and (b) salt‐susceptible accessions. Node size reflects betweenness centrality; node color indicates modularity class; edge thickness scales with correlation strength (|*ρ*|), and edge colors denote positive (red) and negative (green) correlations.

To elucidate how rhizosphere interactions shape salinity responses in soybean, we built genus‐level co‐occurrence networks from shotgun metagenomes of salt‐tolerant and salt‐susceptible accessions. Network metrics underscore a clear contrast between tolerant and susceptible soybeans. The tolerant network contains fewer taxa yet exhibits far more connections, producing a higher mean degree, a shorter characteristic path length, and a smaller diameter (Figure 3a), indicating that its members are more densely interconnected and separated by fewer network steps than those in the susceptible network. The tolerant network positions *Pseudomonas* as the node with the highest betweenness centrality, mirroring recent evidence that soybean roots selectively recruit this genus under saline stress via purine exudation [19]. As core hubs, *Pseudomonas* and *Paenibacillus* include multiple PGPR lineages that produce indole‐3‐acetic acid (IAA) and siderophores under saline conditions [34, 35, 36]. Other prominent hubs include *Pseudorhodoplanes*, *Nitratireductor*, and *Deinococcus*, with the latter being renowned for robust oxidative‐stress defenses [37, 38]. Together, these metrics indicate a more connected, cohesive community with shorter paths between taxa. Such topology is consistent with greater potential for coordinated activity, functional complementarity, and robustness, which may contribute to nutrient turnover, pathogen suppression, and resilience under salt stress.

In contrast, the susceptible network exhibits lower edge density and a longer average path length (Figure 3b), consistent with looser connectivity among genera. Its larger diameter further supports this fragmentation, while the reduced mean degree indicates fewer inter‐taxon associations—features unlikely to support tightly coordinated responses to saline stress. In the susceptible network, *Leptothrix* and *Acinetobacter* form the most cohesive sub‐clusters (Figure 3b), aligning with Leptothrix‐driven Fe(II) oxidation and Fe‐oxyhydroxide sheathing that sequester iron and potentially limit ROS at the root interface [39], together with Acinetobacter EPS–based enhancement of plant salt tolerance [40]. Thus, the prominence of these taxa in susceptible accessions does not necessarily indicate deleterious effects, but may instead reflect the context‐dependent enrichment of taxa with putative local stress‐mitigation functions; however, these functions appear to be confined to fragmented submodules and may be insufficient to establish the more integrated *Pseudomonas*‐centered network associated with tolerant accessions. Although *Azotobacter* and *Ralstonia* rank second and third in betweenness, each connects to only a few neighbors, indicating a keystone‐connector role rather than a classic hub. Overall, the network resembles a patchwork of functional islands rather than an integrated stress‐alleviating consortium, suggesting that beneficial activities may remain confined to smaller sub‐clusters instead of being coordinated across the broader community.

The network view indicates that salt‐tolerant rhizospheres assemble into tightly integrated communities with greater potential for coordinated function, whereas susceptible rhizospheres organize into looser, fragmented modules. This contrast implies differing capacities for nutrient cycling, stress buffering, and pathogen control under salinity.

### 2.4 Correlation Analyses Reveal Rhizobacterial Taxa Linked to Genotype–Dependent Salt Tolerance in Soybean

**FIGURE 4:**
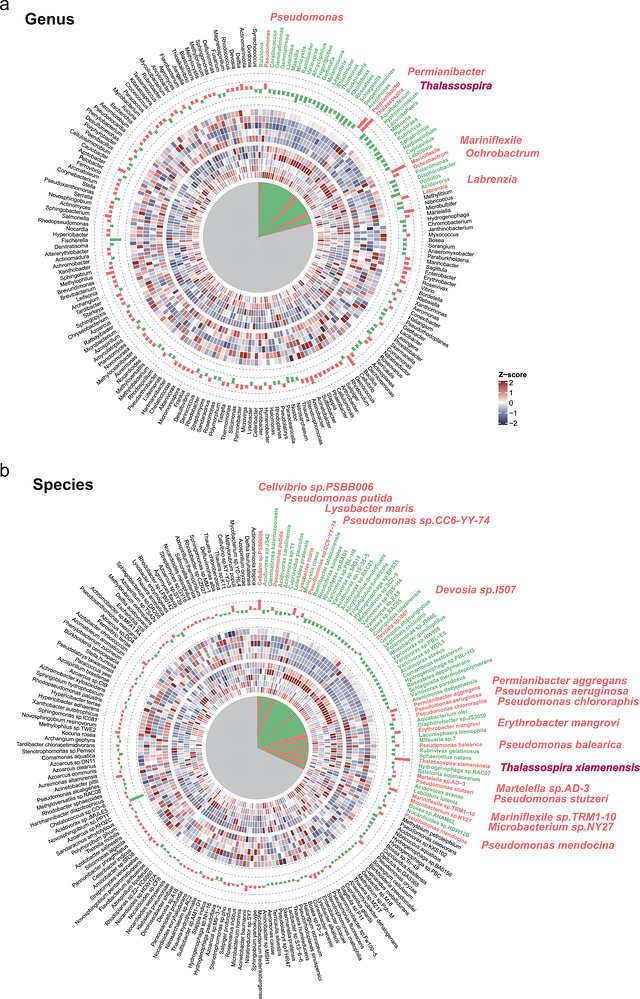
Rhizosphere taxa correlated with soybean salt tolerance across accessions. Circular bar plots with concentric heatmap tracks show associations between rhizosphere microbial taxa and the STI at the genus level (a) and species level (b). Taxon names appear on the outer ring, and the adjacent circular bar for each taxon represents the group‐wise mean abundance ratio between salt‐tolerant and salt‐susceptible soybean accessions (red = enriched in tolerant, bar height = tolerant/susceptible; green = enriched in susceptible, bar height = susceptible/tolerant). Moving inward, successive heatmap rings depict taxon‐wise Z‐score–normalized relative abundance across the ten soybean accessions, ordered outer to inner as: PI 96354, PI 548469, PI 548447, PI 215755, PI 200530, PI 230971, PI 200538, PI 192871, PI 157488, Wm82. For each taxon, positive Z‐scores (red) indicate relatively higher abundance, negative Z‐scores (blue) indicate relatively lower abundance, and values near zero are shown in lighter colors. The innermost ring shows the Spearman correlation between taxon abundance and STI: red, positive correlation (*ρ* > 0, *p* < 0.05); green, negative correlation (*ρ* < 0, *p* < 0.05); gray, not significant (*P* ≥ 0.05).

Guided by evidence that salt tolerance is largely shaped by genotype–microbiota interactions, we next correlated each soybean accession's STI with the relative abundances of individual taxa to identify microbes positively associated with high STI and those linked to diminished tolerance. Among the 200 most abundant rhizosphere genera analyzed, 42 showed significant Spearman correlations with soybean STI (*p* < 0.05) (Figure 4a). Six genera were positively linked to salt tolerance, with Pseudomonas showing the strongest correlation (*ρ* = 0.806, *p* = 0.008). Beyond Pseudomonas, only Ochrobactrum has limited experimental support for enhancing plant salt tolerance [41, 42]. To our knowledge, no experimental reports have yet linked *Thalassospira*, Permianibacter, Mariniflexile, or Labrenzia to enhanced plant salt tolerance, making these genera promising but currently untested candidates. Conversely, 36 genera were negatively associated with soybean salt tolerance, far exceeding the number positively associated. Among them, *Ralstonia* [43], *Burkholderia* [44], *Acidovorax* [45], and *Herbaspirillum* [46] contain confirmed plant‐pathogenic species. Several genera that were more abundant in salt‐tolerant accessions—but whose correlations with STI were not significant (*P* ≥ 0.05)—nonetheless harbor strains with reported salt‐tolerance benefits: Bacillus [47], Enterobacter [48], Streptomyces [49], Klebsiella [50], Arthrobacter [51], Stenotrophomonas [52], Halomonas [53], and Acinetobacter [40]. This may be because the correlations were computed at the genus level, whereas many salt‐tolerance effects are strain‐ or species‐specific; mixing beneficial and non‐beneficial strains within a genus can mask true effects.

Species‐level analyses identified 64 species significantly correlated with soybean STI, including 15 with positive associations (Figure 4b). The strongest correlation was observed for *Cellvibrio* sp. PSBB006 (*ρ* = 0.927, *p* = 0.0001); to our knowledge, neither this taxon nor the genus Cellvibrio has been directly shown in planta to enhance plant salt tolerance. *T. xiamenensis* showed the greatest enrichment in salt‐tolerant vs. susceptible rhizospheres (average fold change = 8.88, *ρ* = 0.397, *p* = 0.031), although its role in the plant rhizosphere remains unclear. Several Pseudomonas species we identified as positively correlated with STI, including P. putida [54], P. stutzeri [55], and P. aeruginosa [56], have been experimentally shown to enhance plant salt tolerance, further supporting the reliability of our correlation analyses. Combining genus‐ and species‐level data, Acidovorax (7 species) and Variovorax (7 species) were uniformly negatively correlated with STI, whereas Pseudomonas (8 species) was uniformly positively correlated, suggesting coherent, genus‐wide patterns that strengthen confidence in these associations. *Acidovorax* is predominantly recognized as a plant pathogen, exemplified by Acidovorax avenae [57], though the genus also harbors commensal and plant‐beneficial members [32]. Variovorax is commonly associated with growth‐supporting functions under non‐stress conditions [58, 59, 60], making its negative association with STI under saline field conditions an unexpected pattern that is considered further in the Discussion.

In summary, our culture‐independent, correlation‐based screen provides a cost‐efficient, field‐anchored screening pipeline that narrows a vast microbial search space to a tractable set of candidates. Using this approach, we identified dozens of rhizosphere taxa newly associated with plant salt tolerance, highlighting promising salt‐tolerant PGPR candidates for experimental validation.

### 2.5 *T. xiamenensis* Alleviates Soybean Salt Stress

**FIGURE 5:**
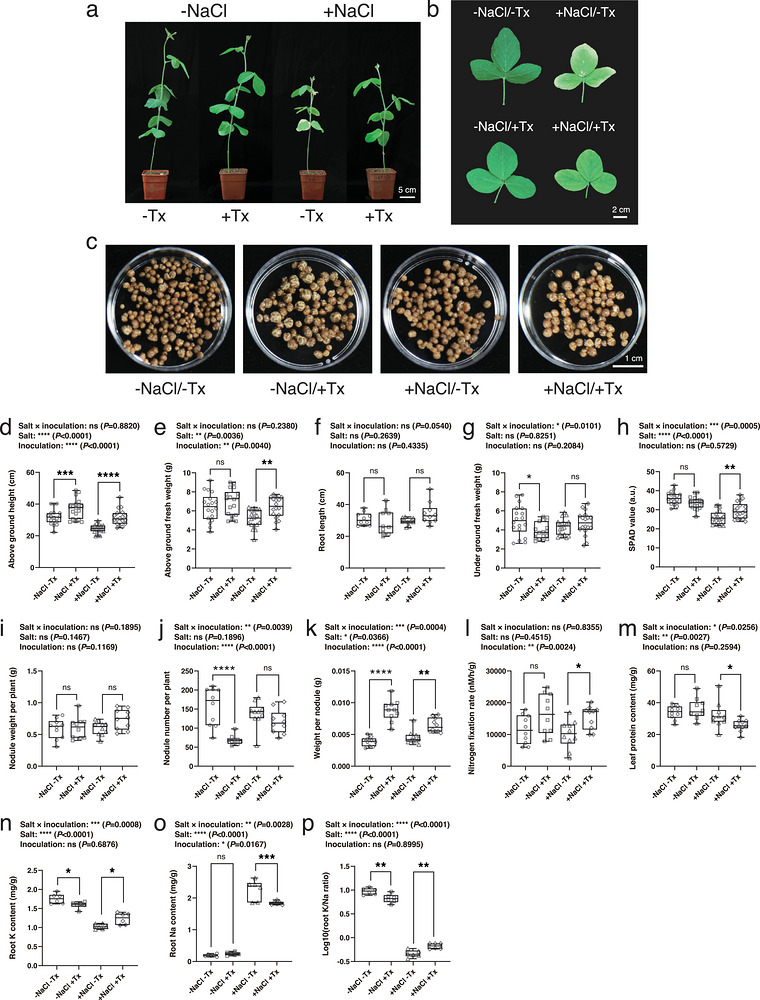
*T. xiamenensis* inoculation enhances soybean salt tolerance in controlled conditions. Soybean (*Glycine max* cv. Wm82) plants were all co‐inoculated with *Bradyrhizobium diazoefficiens* (USDA110) for nodulation, with or without *T. xiamenensis*, and grown in a controlled‐environment chamber either without NaCl or with 50 mm NaCl added. (a) Representative images of whole‐plant phenotypes 28 days post inoculation under four treatments: −NaCl/−Tx, −NaCl/+Tx, +NaCl/−Tx, +NaCl/+Tx (Tx = T. xiamenensis). (b) Close‐ups of basal leaves illustrate chlorosis in +NaCl/−Tx plants and its mitigation by *T. xiamenensis*. (c) Representative whole‐root nodule images from single plants under the four treatments. (d–m) Quantification of phenotypic traits across treatments: shoot height (d) and shoot fresh weight (e), primary root length (f) and root fresh weight (g), SPAD index (h), total nodule fresh weight per plant (i) and nodule number per plant (j), mean fresh weight per nodule (k), nitrogen fixation rate (l), and leaf protein content (m). (n–p) Root ion‐related traits across treatments: root K content (n), root Na content (o), and log10‐transformed root K/Na ratio (p). Data are shown as box‐and‐whisker plots with individual points. Biological replicate numbers per treatment were *n* = 18 for panels d, e, g, and h; *n* = 10 for panels f and i–m; and *n* = 6 for panels n–p. Data were analyzed using ordinary two‐way ANOVA, with salt treatment and *T. xiamenensis* inoculation as fixed factors. The significance of the salt main effect, inoculation main effect, and salt × inoculation interaction is indicated above each panel. Where indicated, Šídák's multiple‐comparison test was used for predefined pairwise comparisons between inoculated and uninoculated plants within each salt condition. (^*^ *p* < 0.05, ^**^ *p* < 0.01, ^***^ *p* < 0.001, ^****^ *p* < 0.0001; ns, not significant). The data shown in d–m are from one representative independent experiment; two additional independent experiments yielded similar trends.

To assess the validity of our correlation results, we evaluated the impact of *T. xiamenensis*, a novel salt‐tolerant PGPR candidate, on soybean responses to salinity. The strain was applied to axenic soybean plants (Wm82) under gnotobiotic conditions. Seedlings were treated with either 50 mm NaCl or water (control), with or without T. xiamenensis inoculation. Under salinity stress, treatment differences were pronounced: inoculated seedlings had greater aboveground height and aboveground fresh weight than uninoculated seedlings (Figure 5a,d,e), whereas root length was not significantly affected by inoculation (Figure 5f,g). In uninoculated plants, the basal compound leaf was chlorotic under salt stress, and this was mitigated by inoculation (Figure 5b). Consistently, the Soil Plant Analysis Development (SPAD) chlorophyll index measured was significantly higher in inoculated plants (Figure 5h), suggesting partial preservation of chlorophyll under salinity. Nodulation traits also responded to inoculation in a trait‐dependent manner. Although total nodule fresh weight per plant was not significantly altered (Figure 5i), inoculation increased mean nodule fresh weight in both non‐saline and saline conditions (Figure 5c,k). Nodule number was reduced by inoculation under non‐saline conditions but was not significantly changed under salinity (Figure 5j). In salinity‐stressed seedlings, inoculation increased nitrogen‐fixation efficiency relative to controls (Figure 5l), whereas leaf protein content showed a salt‐dependent response and was slightly lower in inoculated plants under salinity (Figure 5m). These results suggest that *T. xiamenensis* reshapes nodulation traits under salt stress, favoring larger nodules and enhanced symbiotic performance.

To further evaluate whether *T. xiamenensis* improved ionic homeostasis under salinity, we quantified root K and Na contents. Because excessive Na accumulation is toxic and can interfere with K uptake and retention, whereas K is essential for cellular function, the K/Na ratio is commonly used as an indicator of ionic homeostasis under salt stress. Salt treatment strongly increased root Na accumulation and reduced the root K/Na ratio (Figure 5n–p). Notably, under salinity, *T. xiamenensis* inoculation reduced root Na content and increased root K content, thereby increasing the root K/Na ratio (Figure 5n–p). Consistent with these patterns, two‐way ANOVA revealed significant inoculation effects on aboveground growth and nitrogen‐fixation rate, while SPAD value and root ion‐related traits showed significant salt × inoculation interactions. These results indicate that the protective effect of *T. xiamenensis* was most evident under salinity and was associated with improved chlorophyll status and more favorable root ion balance. Together, these data indicate that *T. xiamenensis* alleviates soybean salt stress by improving aboveground growth, preserving chlorophyll content, enhancing nitrogen‐fixation performance, and partially restoring root ion balance under saline conditions.

### 2.6 *T. xiamenensis* Enhances Soybean Salt Tolerance by Remodeling Transporter and Antioxidant Pathways

**FIGURE 6:**
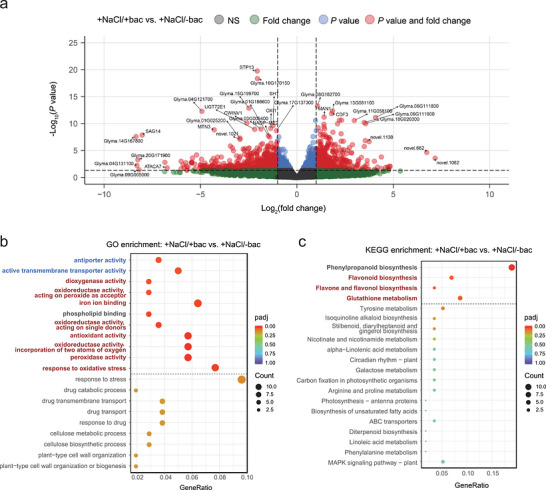
Transcriptomic response of salt‐stressed soybean to *T. xiamenensis* inoculation. (a) Volcano plot showing differential gene expression between *T. xiamenensis–*inoculated and uninoculated soybeans under salt stress. Vertical cutoffs at |log_2_FC| = 1 and a horizontal cutoff at *p* = 0.05. Red points exceed both thresholds; blue are significant with a smaller effect (|log_2_FC| < 1); green show a large effect without significance; and gray are non‐significant. Labels prefixed “novel.” denote transcripts that did not map to the soybean reference genome annotation. (b) GO enrichment of *T. xiamenensis*‐induced/repressed genes reveals over‐representation of antiporter/transporter activities (blue bold), oxidoreductase/peroxidase activity (red bold), and responses to oxidative stress (red bold). (c) KEGG analysis shows enrichment of phenylpropanoid and flavonoid biosynthesis (red bold), glutathione metabolism (red bold), and additional stress‐associated pathways (gray bold). Dashed lines in (b) and (c) denote the significance threshold (BH‐adjusted *p* = 0.05).

To probe how T. xiamenensis mitigates salinity in soybean, we profiled transcripts from inoculated and uninoculated plants under saline and control conditions. Under salinity, inoculated plants showed 578 uniquely expressed genes vs. 958 in uninoculated plants; under control conditions, the counts were 681 and 890, respectively (Figure S3a). Biological triplicates clustered tightly. Unsupervised clustering separated samples primarily by salinity, with a weaker inoculation effect in both environments (Figure S3b), indicating salinity as the dominant source of variance. Differential expression analysis (*p* < 0.05; |log_2_FC| > 1) identified 1666 salt‐responsive differentially expressed genes (DEGs) in uninoculated plants and 250 *T. xiamenensis*‐responsive DEGs under salinity, with the latter comprising 136 upregulated and 114 downregulated genes (Figure 6a and Table S2). Among the 250 DEGs, 144 overlapped with the salt‐responsive set, of which 143 showed opposite expression patterns: 71 were salt‐induced but inoculation‐repressed, whereas 72 were salt‐repressed but inoculation‐induced; only one changed in the same direction (Table S3). This pattern indicates that *T. xiamenensis* primarily reprograms the soybean salt‐response network in an antagonistic manner. Notably, 120 of the 250 DEGs were predicted to encode transporters and 30 were predicted to encode transcription factors (Table S2). Transporter genes comprised a large fraction of DEGs, suggesting that *T. xiamenensis* modulates ion and solute transport to support soybean salt resilience.

GO term enrichment of DEGs between inoculated and uninoculated soybeans under salt conditions showed the strongest signals for antiporter and active transmembrane transporter activities, aligning with the high fraction of transporter‐annotated DEGs. Of the 11 significant terms (*p* < 0.05), 8 related to redox processes, including oxidoreductase activity, iron ion binding, antioxidant activity, and oxidative stress response, while phospholipid binding was also enriched (Figure 6b). Although not statistically significant, several cell wall–related processes ranked among the top 20 GO terms. Overall, the enrichment patterns suggest that T. xiamenensis inoculation under salt stress promotes adaptive rebalancing of ion transport and reinforcement of redox defenses, with phospholipid binding pointing to additional membrane‐associated regulation or signaling. KEGG pathway analysis under salinity showed significant enrichment (*p* < 0.05) of phenylpropanoid and flavonoid biosynthesis, as well as glutathione metabolism between inoculated and uninoculated plants under salinity (Figure 6c). Increased activity of the phenylpropanoid–flavonoid pathways would supply ROS–scavenging metabolites and monolignol precursors for lignification, while the glutathione pathway maintains redox homeostasis and enables glutathione S‐transferase (GST)–mediated detoxification [61, 62, 63, 64], suggesting that inoculation with T. xiamenensis enhances antioxidant defenses and structural resilience under salinity. Together, our results indicate that under salinity, inoculation with T. xiamenensis primarily reprograms ion and solute transport and redox metabolism, with auxiliary membrane‐ and cell wall–associated regulation via phenylpropanoid, flavonoid, and glutathione pathways—a pattern consistent with improved salt tolerance.

### 2.7 Genome Sequencing and Comparative Genomics of *T. xiamenensis* Predict Lineage‐Specific Genes Associated With Rhizosphere Adaptation Under Salinity

**FIGURE 7:**
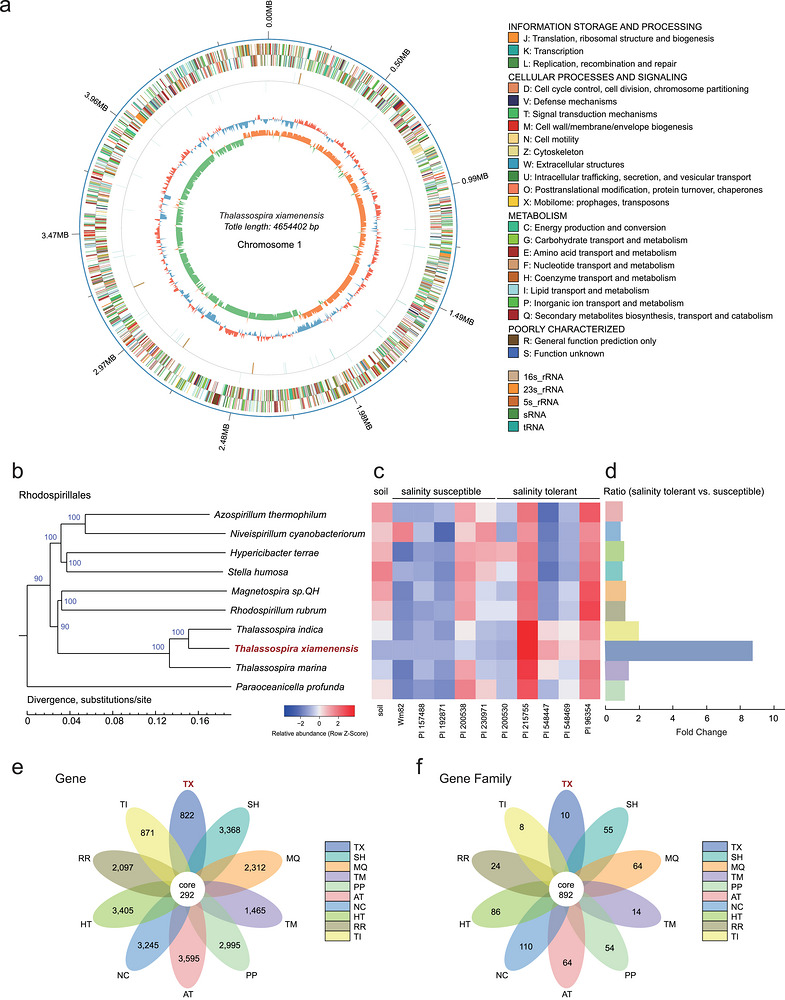
Genome architecture and comparative genomic analysis of *T. xiamenensis*. (a) Circular chromosome map of *T. xiamenensis*. The concentric rings, from the outermost inward, are: (1) Scale ticks marking the genome coordinate scale (Mbp); (2) Protein‐coding genes on the forward and reverse strands, colored according to their COG functional categories (J–S; see key in right panel); (3) Locations of ncRNAs (tRNAs, rRNAs, and sRNAs); (4) GC content: calculated in non‐overlapping windows of (chromosome length/1000) bp. Peaks extending outward (red) indicate regions with GC content above the genomic average, while peaks extending inward (blue) indicate regions below the average. The amplitude corresponds to the degree of deviation from the mean; (5) GC skew: calculated as (G‐C)/(G+C) in non‐overlapping windows of (chromosome length/1000) bp. Peaks extending outward (orange) indicate regions where G > C, while peaks extending inward (green) indicate regions where C > G. The amplitude corresponds to the magnitude of strand asymmetry (GC skew) relative to zero. (b) Core–pan genome phylogeny of ten Rhodospirillales species genomes used for comparison; branch lengths are substitutions per site; node labels indicate bootstrap support (%) from 1000 neighbor‐joining replicates. (c) Heatmap of species‐level relative abundance (row Z‐scores) for Rhodospirillales species across soil and soybean accessions (Wm82, PI 157488, PI 192871, PI 200538, PI 230971, PI 200530, PI 215755, PI 548447, PI 548469, PI 96354). (d) Bars show the fold change in species' relative abundance (tolerant/susceptible), averaged across five salt‐tolerant and five salt‐susceptible accessions. (e) Venn diagram illustrating counts of species‐specific (accessory) and core genes across the ten genomes. (f) Unique vs. core gene‐family counts (orthogroups) counted across the same ten genomes. Species abbreviations: TX (*T. xiamenensis*), TI (*Thalassospira indica*), TM (*Thalassospira marina*), RR (*Rhodospirillum rubrum*), NC (*Niveispirillum cyanobacteriorum*), HT (*Hypericibacter terrae*), SH (*Stella humosa*), MQ (*Magnetospira* sp. QH), PP (*Paraoceanicella profunda*), AT (*Azospirillum thermophilum*).

To clarify its potential contribution to soybean salt tolerance, we sequenced the genome of the *T. xiamenensis* strain used in our validation assays. The assembly yielded a 4 654 412‐bp circular chromosome (54.78% GC) from short reads with an estimated k‐mer coverage of 52.76 **×** (Figure 7a). Functional annotation showed that genes for membrane transport, carbohydrate and energy metabolism, and amino acid metabolism comprise the largest fractions of the *T. xiamenensis* genome (Figure S4a). Secondary‐metabolism mining identified five biosynthetic gene clusters in *T. xiamenensis* (Figure S4b), indicating the capacity to make osmoprotectants and small molecules that support persistence and competition in the rhizosphere. Published evidence indicates that polyketide metabolites, such as those produced by Type I polyketide synthase (T1PKS) pathways, can help plants alleviate salinity stress [49]. In addition, ectoine, a bacterial salt‐protective compatible solute, directly supports osmoprotection in planta [65], while terpene biosynthesis enables production of volatile signals that can prime plant stress responses [66]. The genus *Thalassospira* belongs to the order Rhodospirillales and family Rhodospirillaceae. In addition to *T. xiamenensis*, our shotgun metagenomic sequencing detected nine other Rhodospirillales species, none of which showed significant correlation with STI across accessions (*P* ≥ 0.05). These ten species represented all Rhodospirillales species detected in our metagenomic dataset and were therefore included in the comparative genomic analysis. A core–pan genome phylogeny resolved relationships among ten species, placing T. xiamenensis as sister to T. marina within a clade that also includes T. indica (Figure 7b). Across soybean rhizospheres, these three taxa showed broadly similar abundance patterns relative to the other seven species (Figure 7c). Notably, only *T. xiamenensis* was markedly enriched in salt‐tolerant rhizospheres, with **>**8‐fold abundance difference (Figure 7d).

To characterize genetic determinants potentially related to host salt tolerance, we performed comparative genomics and identified 822 genes unique to *T. xiamenensis* (Figure 7e), including a substantial uncharacterized fraction (*n* = 426). Among T. xiamenensis–specific genes, we found functions suggestive of rhizosphere adaptation to salinity, including transport and nutrient uptake, environmental sensing and motility, secretion and interaction modules, cell‐envelope biogenesis, and stress defense and persistence (Table S4). Gene‐family analysis identified *T. xiamenensis–*specific families, including substrate‐binding proteins, outer‐membrane β‐barrel proteins, DUF697 proteins, and hypothetical proteins (Figure 7f). Taken together, our phylogenomics‐guided comparative genomics identifies lineage‐specific candidate genes in *T. xiamenensis* that may underlie the soybean transcriptomic responses to salinity, providing a mechanistic shortlist for targeted validation and salt‐tolerance inoculant design.

## 3 Discussion

By integrating STI‐based field phenotyping with rhizosphere shotgun metagenomics, we show that soybean salt tolerance in natural saline soils is associated with distinct, host‐linked microbiome configurations. The STI framework is useful because it normalizes plant performance across highly heterogeneous field salinity and thereby provides a practical basis for comparing accessions under realistic coastal conditions. Although STI is derived from survival performance normalized to local salinity, it likely reflects the integrated contribution of multiple physiological determinants of salt tolerance, including ion homeostasis, osmotic adjustment, oxidative‐stress control, and the ability to sustain growth under saline conditions. Coupled with species‐resolved shotgun metagenomics, this framework provides a practical way to move beyond isolate‐based screening and to connect field performance with microbial community composition, functional gene repertoires, and association‐network structure.

Although soil properties, including salinity, can influence rhizosphere community composition and interaction patterns [67, 68], local soil salinity had limited explanatory power in our analyses, whereas host genotype‐associated salt‐tolerance status was strongly associated with the observed differences in dominant genera and co‐occurrence networks. Whether the tolerance‐associated hub taxa and network features identified here are conserved across broader soybean germplasm and other saline soil types remains to be tested. Nevertheless, the prominence of *Pseudomonas* in the tolerant network is consistent with recent evidence that this genus is actively enriched in the roots of salt‐stressed wild soybean [19], supporting the potential contribution of *Pseudomonas*‐rich consortia to soybean salt tolerance. By contrast, *Variovorax* showed a coherent negative association with STI despite its commonly growth‐promoting role under non‐stress conditions. *Variovorax* is known to modulate plant hormone balance by degrading microbe‐derived auxin and lowering ethylene through ACC deaminase activity, thereby restoring root growth under non‐stress conditions [58, 59, 60]. Under salinity, however, plants often rely on elevated ethylene signaling and finely tuned auxin gradients to trigger protective responses, and suppression of ethylene signaling can increase salt sensitivity [69]. Salt stress also commonly inhibits primary root growth and remodels lateral root development through changes in auxin distribution and signaling [70, 71, 72]. In this context, a *Variovorax* population that persistently removes auxin and reduces ethylene could oppose these adaptive responses, maintaining growth‐promoting signals when restrained growth and root architectural adjustment would be more beneficial. This interpretation suggests that a typically beneficial rhizobacterial genus may shift from a growth‐supporting to a stress‐compromising role under saline field conditions, underscoring the environment‐dependent nature of PGPR effects. Correlation analysis also recovered known salt‐tolerant PGPR and identified novel taxa, including T. xiamenensis, whose growth‐promoting effect under salinity was confirmed experimentally. Together with the accompanying RNA‐seq results implicating ion transport and redox homeostasis, two processes central to plant salt tolerance [73], these findings support the utility of our framework for identifying microbiome features and candidate taxa linked to soybean salt tolerance in natural saline soils.

While this framework advances field‐based discovery of salt‐tolerance–promoting microbes, it also has a defined scope and several important considerations. By design, our genotype‐centric strategy detects microbes whose abundance differences are driven by host genotype; thus, broadly beneficial taxa that all soybean genotypes attract or repel similarly would escape detection. Because our analyses rely on reference genomes, taxa that currently lack representative genomes in public databases remain unresolved at the species level and are not yet captured as explicit candidate beneficial microbes, even though they may contribute to salt tolerance. As with any correlation‐based framework, the associations we observe do not on their own establish causality: microbes enriched in salt‐tolerant accessions may be drivers of tolerance, passengers favored by tolerant genotypes, or responders to host physiological state. Each candidate species identified here ultimately requires experimental validation. In practical terms, strains that can be isolated or accessed as pure cultures are the most immediate targets for phenotypic testing and application, and different strains within the same species may exert non‐equivalent effects on the host.

Looking forward, a critical question is how soybean genotypes shape the specificity of their rhizosphere microbiome. Comparative genomic or association analyses between tolerant and susceptible accessions may pinpoint loci underlying such differences and illuminate how certain hosts recruit beneficial microbes to enhance salinity tolerance. Many small‐effect alleles, often related to root exudation, hormone signaling, or immunity, have been found to modulate the root microbiome [74]. In parallel, an important next step is to elucidate the molecular dialogue between soybean and its microbiome under salinity. In salt‐stressed plants inoculated with *T. xiamenensis* vs. uninoculated controls, functional validation of DEGs is essential. Additionally, untargeted and stable‐isotope–resolved metabolomics and exometabolomics, combined with bioassay‐guided fractionation and chemoproteomic pull‐downs, can uncover the microbial signals that trigger ion‐transport and redox programs during salt stress. The correlation analyses shown in Figure 4 focused on the top 200 genera and top 200 species, although many low‐abundance bacterial taxa were also significantly enriched in salt‐tolerant rhizospheres. While the top taxa were prioritized in this first‐pass correlation screen because they are more likely to represent biologically important core members of the soybean rhizosphere community and dominant contributors to community structure and function, potentially important rare taxa were not systematically explored in the present study. These rare taxa, together with additional salt‐tolerance‐associated PGPR candidates within the top 200 that were not functionally validated here, will be important subjects for future study. By assembling a synthetic community (SynCom) from co‐occurrence‐network hub taxa and taxa significantly correlated with the STI, researchers can experimentally validate community‐level effects on plant performance and develop inoculants that outperform single‐strain PGPR.

Beyond soybeans, the approach could be adapted to other crop species to identify microbiome mediators of stress tolerance. Integrating STI–guided association mapping with species‐resolved metagenomics and targeted functional assays accelerates the identification of high‐confidence candidates, while providing a tractable route to mechanistic validation and field‐ready inoculants and breeding markers.

## 4 Experimental Section

### 4.1 Plant Growth and Salt‐Tolerant Screening

All soybean accessions were sourced from the US Department of Agriculture soybean germplasm collection, except *Williams 82* (Wm82). Soybean seeds were germinated under greenhouse conditions and, at 7 days after emergence, transplanted to a saline field in Dongying of the Yellow River Delta (YRD), China. Seedlings were established at 30 cm within‐ and between‐row spacing; for each accession, around 40 plants were set in square block plots. At 20 days post‐transplant, plant survival was recorded, and rhizosphere salinity was measured in situ with a PNT3000 activity meter (STEP GmbH, Nürnberg, Germany). For each accession, one salinity reading was taken for every surviving and every dead plant, ∼5 cm from the primary root at 7–10 cm depth. The instrument was operated over its 0–10 activity‐unit range (resolution 0.01 g L^−^^1^). Survival rate (%) = (number of surviving plants / total transplanted) × 100. Mean accession rhizosphere salinity = (sum of salinity for surviving plants + sum for dead plants) / number of transplanted plants.

### 4.2 Rhizosphere and Bulk Soil DNA Isolation

Intact soybean roots were collected, shaken to remove loosely adhering soil, and immediately kept on ice. For each accession, roots from five individual plants (five biological replicates) were immersed in pre‐chilled PBS (pH 7.4; Gibco, 10010023) and vortexed at full speed for 15 s; the buffer was then replaced, and the roots were vortexed again. The two washes from the same root were pooled and passed through a 100 µm sterile strainer (Falcon, 352360), and the flow‐through was centrifuged at 6000 × g for 20 min at 4°C to pellet the rhizosphere fraction. Bulk soil was sampled ∼20 cm laterally from the nearest root system at 0–15 cm depth using a sterile soil corer. DNA was extracted from rhizosphere pellets using the DNeasy PowerSoil Pro (QIAGEN, 47014) and from bulk soil using DNeasy PowerMax Soil (QIAGEN, 12988‐10) according to kit manuals. DNA yield and purity were assessed with NanoDrop 2000 (Thermo Scientific, ND‐2000).

### 4.3 Shotgun Metagenomic Profiling

For each soybean accession, rhizosphere DNA samples that passed quality control (agarose‐gel integrity without smear; total amount ≥ 1 µg; A260/280 = 1.8–2.0; A260/230> 1.8) were retained. Three bulk‐soil DNA samples passing quality control were randomly selected for sequencing as environmental references. Replicate‐level libraries were constructed and sequenced for Wm82 and the five salt‐tolerant accessions (triplicate libraries per accession). For the four salt‐susceptible accessions, equal‐mass aliquots from the three replicates were pooled within each accession to produce a single library per accession prior to sequencing. All statistical tests in this study were conducted at the accession level, using the mean of tolerant triplicates and a single pooled value for susceptible accessions. This asymmetric design concentrates replication in the group of primary interest (salt‐tolerant accessions) and includes a post hoc check that tolerance‐enriched taxa were consistent across tolerant triplicates. For library construction, 1 µg genomic DNA per sample was processed with the MGIEasy Universal DNA Library Prep Set (MGI‐Shenzhen, China) with bead‐based size selection using MGIEasy DNA Clean Beads (MGI‐Shenzhen, China), followed by dsDNA denaturation and circularization with the MGIEasy Circularization Module V2.0 (MGI‐Shenzhen, China) to generate single‐stranded circular templates for DNA nanoball formation; libraries were sequenced as paired‐end 150 bp (PE150) on DNBSEQ‐T7 platform (MGI‐Shenzhen, China). Raw reads were quality‐filtered with SOAPnuke v2.2.1 [75], and host reads were removed by aligning to the Glycine max reference genome with Bowtie2 v2.2.5 [76], and high‐quality reads de novo assembled with MEGAHIT [77]; contigs < 300 bp were discarded. Genes were predicted with MetaGeneMark [78] and clustered with CD‐HIT [79] (95% identity; 90% coverage) to generate a nonredundant catalogue. Gene abundances were estimated with Salmon [80]. Functional annotation used DIAMOND [81] against KEGG, eggNOG, COG, UniProt, CAZy, BacMet and CARD. Taxonomic classification used Kraken 2 [82] with abundance re‐estimation by Bracken [83]. The principal soybean rhizobial genera (*Sinorhizobium*, *Ensifer*, *Rhizobium*, *Bradyrhizobium*, and *Mesorhizobium*), together with *Agrobacterium*, were excluded from the abundance tables before downstream analyses. The relative abundances of the remaining taxa were then renormalized to 100% for downstream co‐occurrence and correlation analyses. Between‐group comparisons used Wilcoxon rank‐sum (2 groups) or Kruskal–Wallis (≥ 3 groups) with Benjamini–Hochberg false discovery rate (BH FDR) control (*q* < 0.05).

### 4.4 Co‐Occurrence Network Analysis

Analyses were performed separately for tolerant and susceptible groups (*n* = 5 accessions each). Within each group, we retained genera detected in all five accessions (100% prevalence) and with a cumulative relative abundance ≥ 0.005. Then, the Spearman correlation coefficients between microbial genera were calculated using Hmisc::rcorr in R to assess the correlation of their abundance changes [84]. To ensure the significance of network relationships, we applied a correlation threshold (|*ρ*| ≥ 0.7) and required BH‐adjusted *p* < 0.05 to control for multiple testing. By combining the correlation coefficients with the significance level, an adjacency matrix was generated, and self‐loops (autocorrelations) were removed, ultimately obtaining the co‐occurrence network between microbial genera. The igraph package was then used to convert the adjacency matrix into a weighted network [85], where the edge weight represents the Spearman correlation coefficient between microbial genera. To simplify the network structure, isolated nodes (degree = 0) were removed. After filtering and removal of isolated nodes, the final networks contained 167 genera in the tolerant group and 239 genera in the susceptible group. Network visualization was first generated in R, and the adjacency matrix, edge list, and node attribute table were exported for further layout and editing in Gephi [86].

### 4.5 Controlled‐Environment Salt‐Stress Phenotyping of Soybean

Seeds of *Glycine max* cv. Wm82 were surface‐sterilized by 70% (v/v) ethanol for 1–2 min, followed by 1% (w/v) available chlorine (from NaOCl stock diluted to 1%) for 10 min, and rinsed 5× with sterile distilled water. Sterile vermiculite (autoclaved) was used as substrate. Seedlings were grown under a 16 h light/8 h dark photoperiod at a constant temperature of 28°C. The experiment comprised four treatments with 18 seedlings each: (i) 0 mm added NaCl; (ii) 0 mm added NaCl plus T. xiamenensis; (iii) 50 mm added NaCl; and (iv) 50 mm added NaCl plus T. xiamenensis. The number of biological replicates used varied among analyses, as indicated in the corresponding figure legends. Salt stress was applied from day 5 post‐germination onward by irrigating exclusively with nitrogen‐free Hoagland solution (per liter: 98.6 mg MgSO_4_·7H_2_O, 69.7 mg K_2_SO_4_, 117.6 mg CaCl_2_·2H_2_O, 34.8 mg K_2_HPO_4_, 0.711 mg H_3_BO_3_, 0.445 mg MnCl_2_·4H_2_O, 0.037 mg CuSO_4_·5H_2_O, 0.102 mg ZnCl_2_, 0.012 mg Na_2_MoO_4_·2H_2_O, 25 mg FeSO_4_·7H_2_O, 33.5 mg EDTA‐2Na) containing either no additional NaCl supplementation or 50 mm added NaCl and sterile water; leachate electrical conductivity (EC) was monitored to maintain target salinity. Bradyrhizobium diazoefficiens USDA110 was cultured in yeast mannitol medium (10 g L^−^^1^ mannitol, 3 g L^−^^1^ yeast extract, 0.2 g L^−^^1^ MgSO_4_·7H_2_O, 0.1 g L^−^^1^ NaCl, 0.25 g L^−^^1^ K_2_HPO_4_, 0.25 g L^−^^1^ KH_2_PO_4_; pH 6.8–7.0) at 28°C. Thalassospira xiamenensis strain TX (CGMCC 1.8609; original strain ZJ1259), originally isolated from seawater and obtained as a lyophilized culture from the China General Microbiological Culture Collection Center. T. xiamenensis was cultured at 28°C in a marine‐salt medium (per liter: 5.0 g peptone, 1.0 g yeast extract, 0.1 g ferric citrate, 19.45 g NaCl, 5.9 g MgCl_2_, 3.24 g Na_2_SO_4_, 1.8 g CaCl_2_, 0.55 g KCl, 0.16 g NaHCO_3_, 0.08 g NaBr, 0.034 g SrCl_2_, 0.022 g H_3_BO_3_, 0.004 g Na_2_SiO_3_, 0.0024 g NaF, 0.0016 g NH_4_NO_3_, 0.008 g Na_2_HPO_4_; pH 7.4–7.8). To establish nodulation, at 7 days post‐germination all plants were inoculated at the root zone with USDA110 (OD_600_ = 0.02; 50 mL per plant). Where indicated, T. xiamenensis was co‐inoculated at OD_600_ = 0.02 (50 mL per plant); controls received equal volumes of sterile medium. At 28 days after USDA110 inoculation, shoot height and fresh weight, primary root length and root fresh weight, SPAD values, nodule number and total nodule fresh weight per plant, mean fresh weight per nodule, nitrogen fixation rate, and leaf protein content were determined using standard procedures described with the results. Nitrogen fixation was assessed with the acetylene reduction assay (ARA) (after evacuating, 10% acetylene in vial headspace; incubation 60 min at 25°C) following the published procedures [87], and the ethylene production was quantified by gas chromatography (ShangHaiSePu, C9310). Chlorophyll content was estimated as the SPAD index with a SPAD‐502Plus chlorophyll meter (Konica Minolta, Osaka, Japan). For each plant, three readings were taken on the second youngest fully expanded leaf, then averaged. Leaf protein was quantified using the bicinchoninic acid (BCA) assay from 100 mg of fully expanded leaves. Tissue was flash‐frozen, ground in liquid N_2_, and extracted in ice‐cold buffer (50 mm Tris‐HCl, pH 7.5, 150 mm NaCl, 1 mm ethylenediaminetetraacetic acid, 1% (w/v) polyvinylpolypyrrolidone, 1% (v/v) protease inhibitor cocktail (Sigma, P9599)). Clarified extracts (12 000 × g, 10 min, 4°C) were assayed with a BCA kit (Thermo Fisher Scientific, 23227) against bovine serum albumin (BSA) standards. After 30 min at 37°C, A_562_ was read and protein reported as mg g^−^^1^ fresh weight.

### 4.6 Ion Content Measurements

Root Na and K contents were determined using soybean plants from four treatment groups: (i) 0 mm added NaCl; (ii) 0 mm added NaCl plus *T. xiamenensis*; (iii) 50 mm added NaCl; and (iv) 50 mm added NaCl plus *T. xiamenensis*, with six biological replicates per group. For each biological replicate, approximately 1 g of fresh root tissue was collected for total Na and K quantification. Root samples were homogenized, and acid‐digested before elemental analysis. Briefly, prepared root samples were digested with a nitric acid–perchloric acid mixture, and reagent blanks were included for background correction. Total Na and K contents were quantified using flame photometry against corresponding Na and K standard curves, following established methods for plant ion analysis [88]. Ion contents were calculated based on the measured concentration in the digest, reagent blank correction, final volume, dilution factor, and sample weight.

### 4.7 Transcriptome Sequencing and Functional Interpretation

RNA‐seq was performed for four conditions: (i) 0 mm added NaCl, (ii) 0 mm added NaCl + *T. xiamenensis*, (iii) 50 mm added NaCl, and (iv) 50 mm added NaCl + *T. xiamenensis*; with three biological replicates (individual plants) per condition. Total RNA was extracted from 28 days after germination soybean roots with the RNAprep Pure Plant Plus Kit (Polysaccharide & Polyphenol‐rich) (TIANGEN, DP441) following the manufacturer's protocol. RNA integrity and size distribution were assessed on an Agilent 2100 Bioanalyzer (Agilent, model G2939BA). Poly(A)+ mRNA was enriched with oligo(dT) magnetic beads, fragmented, and reverse‐transcribed using random hexamers. After end repair, A‐tailing, adaptor ligation, size selection, PCR amplification, and purification, libraries were prepared with Fast RNA‐seq Lib Prep Kit V2 (ABclonal, RK20306). Passing libraries were quantified by Qubit and qPCR and checked on the Bioanalyzer, then pooled and sequenced (paired‐end 2 × 150 bp) on an Illumina NovaSeq X Plus instrument. Reads were quality‐filtered with fastp [89], mapped to the Wm82 reference genome (a4.v1) with corresponding GTF annotation using HISAT2 v2.0.5 [90], and assembled with StringTie [91]. Gene‐level counts were obtained by featureCounts [92]; expression was summarized as FPKM. All differential expression analyses were performed with DESeq2 on raw counts [93]. Transporters and transcription factors among DEGs were annotated using TrSSP [94] and PlantTFDB [95], respectively. Functional enrichment analyses for GO and KEGG pathways were performed using clusterProfiler [96].

### 4.8 Genome Sequencing

T. xiamenensis (CGMCC 1.8609) genomic DNA was extracted by saline Tris‐EDTA (STE) lysis. DNA integrity was checked by agarose gel electrophoresis, and concentrations were measured with a Qubit 4 Fluorometer (Thermo Fisher Scientific, Q33226). Genomic DNA was fragmented to 6–20 kb with a g‐TUBE (Covaris, 520079), cleaned with AMPure PB beads (PacBio, 100‐265‐900), and libraries were prepared with the SMRTbell Express Template Preparation Kit 2.0 (PacBio, 100‐938‐900). Sequencing primer annealing and Sequel II polymerase binding followed the manufacturer's instructions, and complexes were loaded onto SMRT Cell 8 m consumables (PacBio, 101‐389‐001) and sequenced with the Sequel II Sequencing Kit 2.0 (PacBio, 101‐820‐200) on a Sequel II System operating zero‐mode waveguide (ZMW) optics.

### 4.9 Genome Assembly, Annotation, and Functional Analyses

Long‐read data were assembled with Canu v2.0 [97] and polished in three rounds with Racon v1.4.13 [98], followed by three short‐read polishing rounds with Pilon v1.22 [99] to obtain the final consensus. Open reading frames were predicted from contigs ≥500 bp using GeneMarkS for prokaryotic genomes [100]. For functional annotation, predicted proteins were first searched with sequence aligner BLASTP against UniProt and NCBI RefSeq (retaining matches with E‐value ≤ 1e−5, identity ≥ 40%, and bidirectional coverage ≥ 60%). Orthology‐based functional terms were assigned using eggNOG‐mapper to propagate GO annotations and KEGG Orthology identifiers. Secondary‐metabolite biosynthetic gene clusters were identified using antiSMASH v2.0.2 [101].

### 4.10 Comparative Genomic Analysis

Pairwise and multi‐genome comparisons were performed using MUMmer [102] and LASTZ [103], and syntenic blocks were inferred from the resulting alignments. Core genes and specific genes were identified with CD‐HIT (50% pairwise identity; 0.7 alignment‐coverage threshold) [79]. Genes present in all genomes were designated core, whereas those restricted to a single genome were designated specific; all remaining non‐universal genes were considered dispensable. The pan‐genome comprised the union of core and dispensable genes. Gene families were constructed using BLAST [104], followed by consolidation of overlapping high‐scoring segment pairs and applying hierarchical clustering with hcluster_sg [105, 106], as implemented in TreeFam/Ensembl‐style workflows. Single‐copy core orthologs were aligned using MUSCLE [107], and phylogenetic reconstruction employed TreeBeST under neighbor‐joining with 1000 bootstrap replicates [105]. Resulting overlaps among core and specific sets across genomes were visualized with Venn diagrams.

### 4.11 Statistical Analysis

Phenotypic data from greenhouse inoculation assays were analyzed using GraphPad Prism 10.1.2. Data were analyzed by ordinary two‐way ANOVA, with salt treatment and bacterial inoculation as fixed factors. Data shown in Figure 5d–m were from one representative independent experiment, while two additional independent experiments yielded similar trends (Table S5). For each trait, the significance of the salt main effect, inoculation main effect, and salt × inoculation interaction was evaluated. Where indicated, predefined pairwise comparisons between inoculated and uninoculated plants within each salt condition were performed using Šídák's multiple‐comparison test. Differences were considered statistically significant at *p* < 0.05.

## Author Contributions

**Yu Luo** conceived the study. **Wei‐Cai Yang** provided overall supervision, resources, and project support. **Yu Luo** designed the experiments. **Feng‐Li Kang**, **Qi‐Min Li**, and **Yu Luo** performed the experiments. **Yu Luo** analyzed the data, conducted bioinformatics and statistical analyses, and wrote the manuscript.

## Funding

This work was supported by the CAS Project for the Young Scientists in Basic Research (Grant No. YSBR‐011 to Y.L.), the National Key Research and Development Program of China (Grant No. 2023YFD1200600 to Y.L.), the Strategic Priority Research Program of the Chinese Academy of Sciences (Grant No. XDA24010205 to W.C.Y.; Grant No. XDA26030105 to Y.L.), and the Young Elite Scientists Sponsorship Program by CAST (Grant No. 2016QNRC001 to Y.L.).

## Conflicts of Interest

Patent applications entitled “Systems and methods for screening microbial taxa that enhance plant salt tolerance” (202511608085.6) and “*Thalassospira xiamenensis–*based biological preparations for increasing soybean salt tolerance” (202511319033.7) have been filed in China and are pending. Y.L., W.‐C.Y., and F.‐L.K. are listed as inventors. The authors declare no other competing interests.

## Supporting information




**Supporting File 1**: advs76373‐sup‐0001‐FigureS1‐S4.pdf.

**Supporting File 2**: advs76373‐sup‐0002‐TableS1.xlsx.

**Supporting File 3**: advs76373‐sup‐0003‐TableS2.xlsx.

**Supporting File 4**: advs76373‐sup‐0004‐TableS3.xlsx.

**Supporting File 5**: advs76373‐sup‐0005‐TableS4.xlsx.

**Supporting File 6**: advs76373‐sup‐0006‐TableS5.xlsx.

## Data Availability

All raw sequencing data have been deposited in the NCBI Sequence Read Archive (SRA) under BioProject PRJNA1370302 and SRA Study SRP649515. This Study comprises three data types: shotgun metagenomes from rhizosphere and bulk soil samples, whole‐genome sequencing of *Thalassospira xiamenensis*, and root RNA‐seq of *Glycine max*.
