# Supplementary material for: Metagenomic Association Uncovers Host Genotype‐Structured Rhizobacterial Networks and Novel Taxa That Enhance Soybean Salt Tolerance
Source: Adv Sci (Weinh). 2026 Jul 13;13(53):e76373. doi: 10.1002/advs.76373 (PMC13360111; doi:10.1002/advs.76373)
Supplement: Supplementary file 1 [file ADVS-13-e76373-s005.pdf]

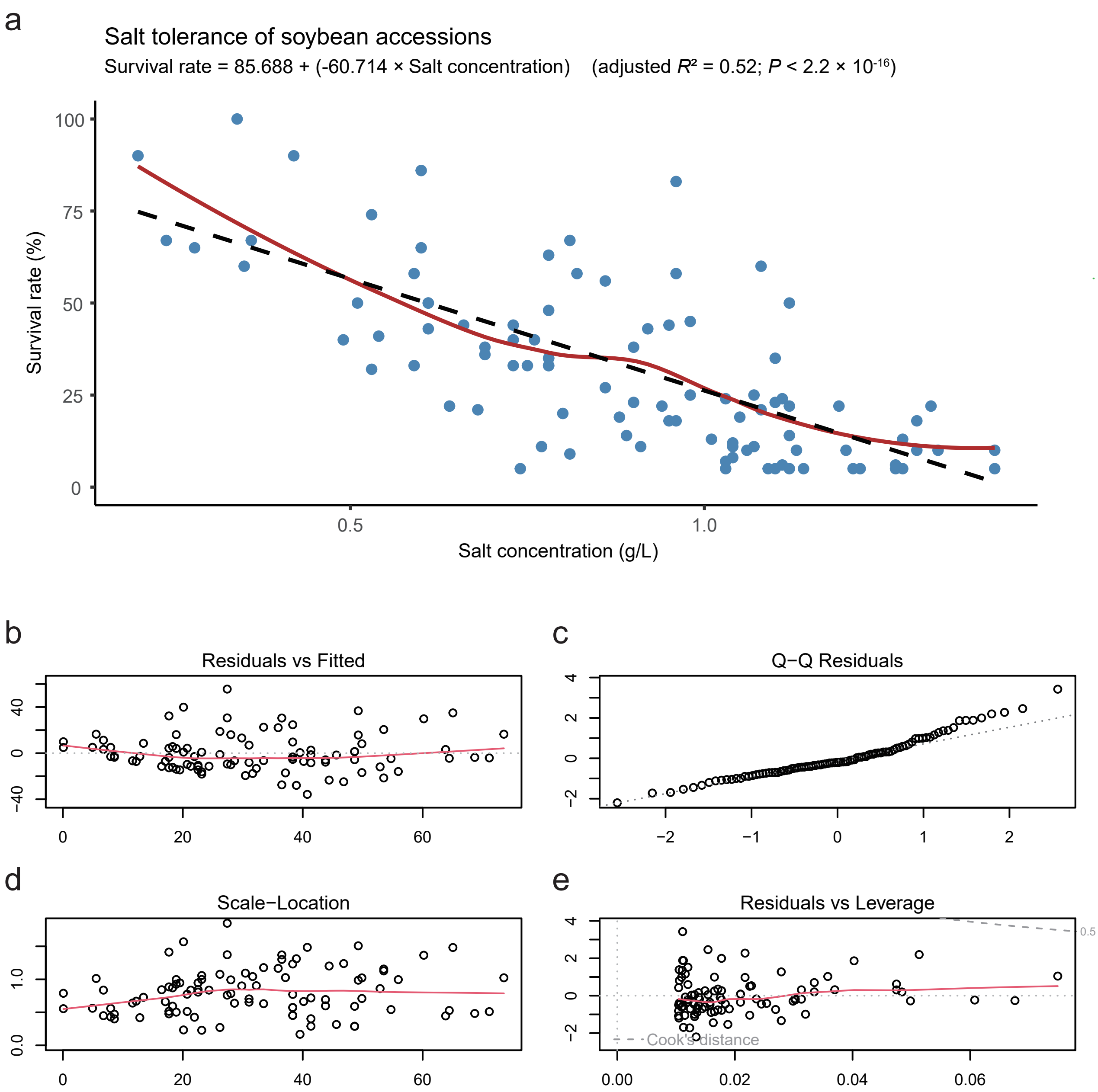

Supplementary Figure 1. Survival–salinity relationship and linear-model diagnostics. (a) Scatter-plot of soybean accession survival rate versus local soil salt concentration (g/L) with the fitted ordinary least-squares (OLS) regression line and a nonparametric LOESS smoother; accessions exhibiting complete mortality were excluded to avoid zero-variance artifacts. (b-e) Graphical diagnostics for the OLS fit. (b) Residuals versus fitted values show random dispersion, consistent with linearity. (c) Q–Q plot of residuals suggests approximately normal errors with only mild tail departures. (d) Scale-location plot shows stable spread of standardized residuals, consistent with homoscedasticity (Breusch-Pagan non-significant). (e) Residuals versus leverage indicates the absence of high-influence cases; all points lie within conservative Cook's-distance bounds. Agree-ment between LOESS and OLS, together with the diagnostic outcomes, supports the use of a simple linear specification.

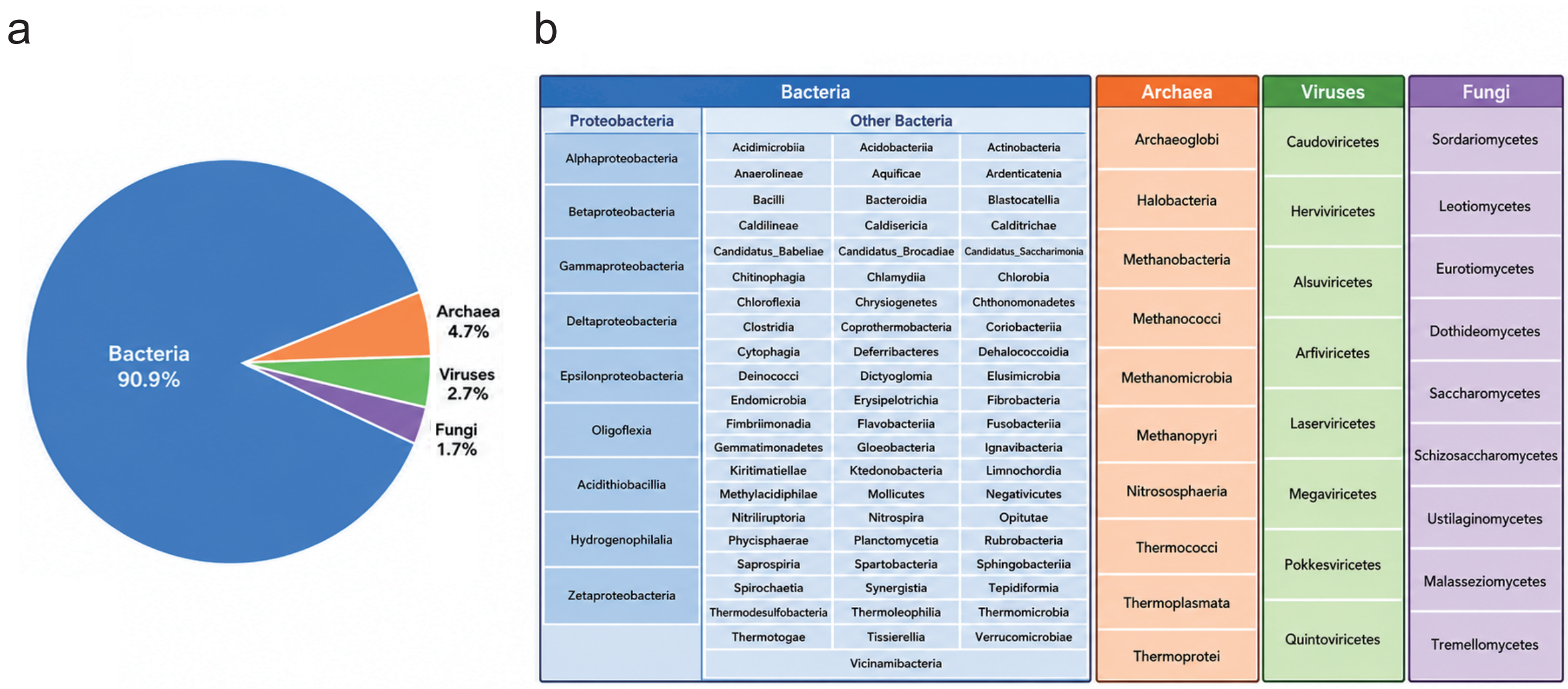

Supplementary Figure 2. Taxonomic composition and class-level classification of taxa detected across bulk soil and rhizosphere metagenomes. (a) Pie chart showing the relative proportions of the taxa assigned to Bacteria, Archaea, Viruses, and Fungi across bulk soil and rhizosphere metag-enomes. (b) The class-level taxonomic classification of the detected taxa. Colors correspond to the major taxonomic groups: blue, Bacteria; orange, Archaea; green, Viruses; and purple, Fungi.

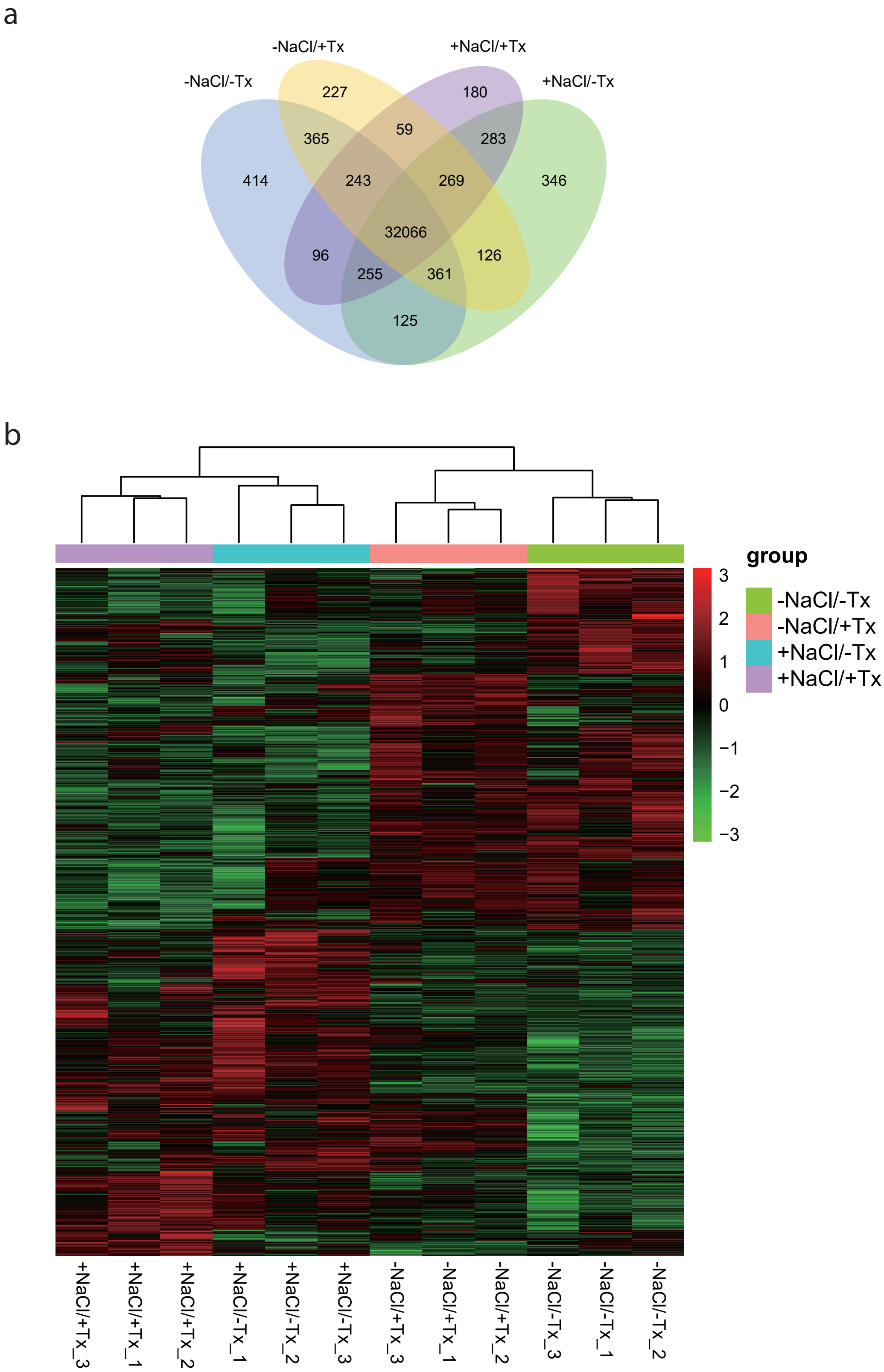

Supplementary Figure 3. Global differential expression across *T. xiamenensis* inoculation and salinity treatments. Soybean roots were sampled under four conditions: mock and *T. xiamenensis*-inoculated, each with or without 50 mM NaCl added (n = 3 independent biological replicates per condition). RNA-seq, read processing, normalization, and differential analyses were performed as detailed in Methods. (a) Venn diagram summarizing specifically and commonly expressed genes across four conditions, highlighting shared versus condition-specific regulation. (b) Heatmap of DEG expression (row-centered and scaled) with unsupervised hierarchical clustering of genes and samples. Replicate samples cluster within conditions, and conditions separate according to inoculation and salt status. Color scale represents relative expression (z-scores).

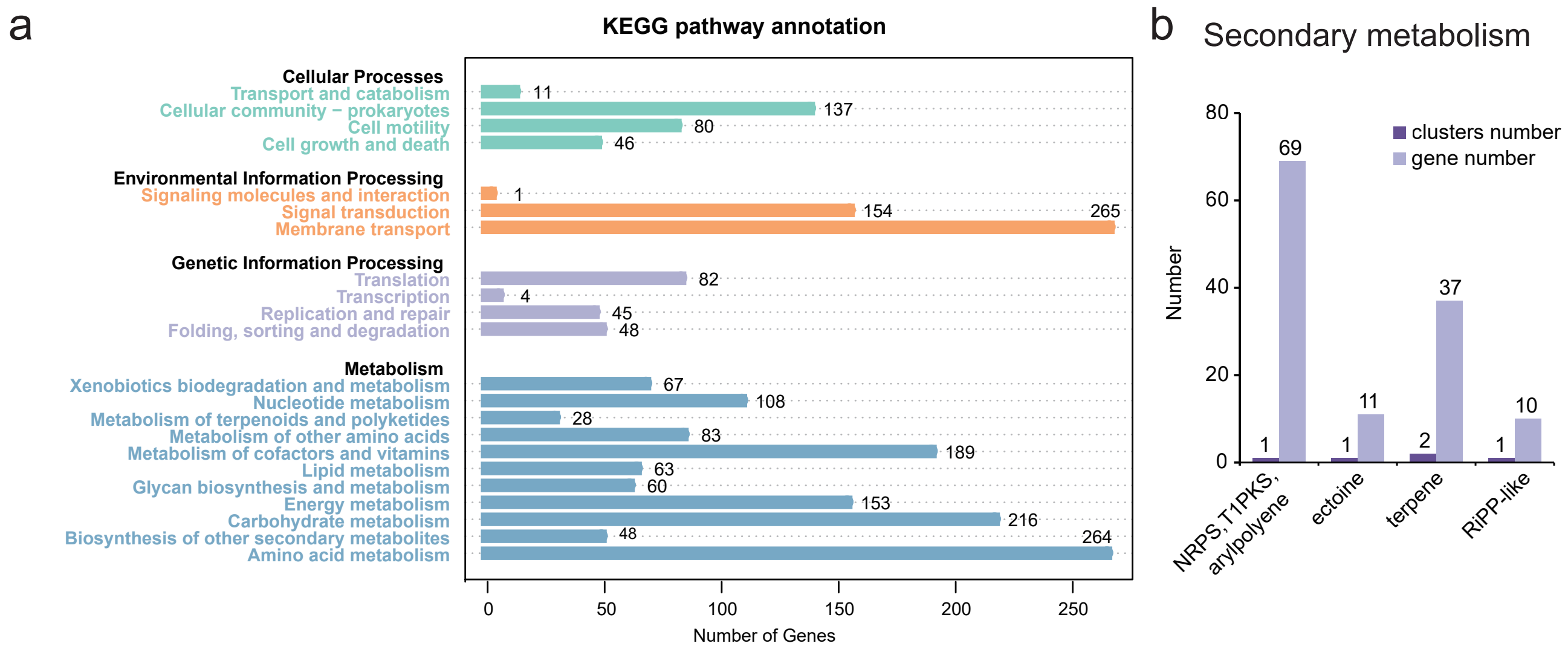

Supplementary Figure 4. KEGG functions and specialized metabolic capacity of *T. xiamenensis*. (a) *T. xiamenensis* genes were annotated using KEGG Orthology. Horizontal bar length is propor-tional to the number of genes assigned to each KEGG pathway. (b) Predicted secondary-metabolite biosynthetic gene clusters are grouped by class; dark bars indicate cluster counts, and light bars indicate the constituent genes.
